# Stratification of chemotherapy-treated stage III colorectal cancer patients using multiplexed imaging and single-cell analysis of T-cell populations

**DOI:** 10.1038/s41379-021-00953-0

**Published:** 2021-11-03

**Authors:** Xanthi Stachtea, Maurice B. Loughrey, Manuela Salvucci, Andreas U. Lindner, Sanghee Cho, Elizabeth McDonough, Anup Sood, John Graf, Alberto Santamaria-Pang, Alex Corwin, Pierre Laurent-Puig, Sonali Dasgupta, Jinru Shia, Jonathan R. Owens, Samantha Abate, Sandra Van Schaeybroeck, Mark Lawler, Jochen H. M. Prehn, Fiona Ginty, Daniel B. Longley

**Affiliations:** 1 Patrick G. Johnston Centre for Cancer Research, School of Medicine, Dentistry and Biomedical Science, Queen’s University Belfast, Northern Ireland, UK; 2 Department of Cellular Pathology, Royal Victoria Hospital, Belfast Health and Social Care trust, Belfast, UK; 3 Department of Physiology and Medical Physics and Centre for Systems Medicine, Royal College of Surgeons in Ireland (RCSI) University of Medicine and Health Sciences, 123 St. Stephen’s Green, Dublin 2, Ireland; 4 GE Research Center, 1 Research Circle, Niskayuna, NY 12309 USA; 5 UMR-S 1147, Université Paris Descartes, Paris, France; 6 Velindre Cancer Centre, Velindre Rd, Cardiff, UK; 7 Memorial Sloan Kettering Cancer Center, New York, NY USA

**Keywords:** Colon cancer, Cancer microenvironment

## Abstract

Colorectal cancer (CRC) has one of the highest cancer incidences and mortality rates. In stage III, postoperative chemotherapy benefits <20% of patients, while more than 50% will develop distant metastases. Biomarkers for identification of patients at increased risk of disease recurrence following adjuvant chemotherapy are currently lacking. In this study, we assessed immune signatures in the tumor and tumor microenvironment (TME) using an in situ multiplexed immunofluorescence imaging and single-cell analysis technology (Cell DIVE^TM^) and evaluated their correlations with patient outcomes. Tissue microarrays (TMAs) with up to three 1 mm diameter cores per patient were prepared from 117 stage III CRC patients treated with adjuvant fluoropyrimidine/oxaliplatin (FOLFOX) chemotherapy. Single sections underwent multiplexed immunofluorescence staining for immune cell markers (CD45, CD3, CD4, CD8, FOXP3, PD1) and tumor/cell segmentation markers (DAPI, pan-cytokeratin, AE1, NaKATPase, and S6). We used annotations and a probabilistic classification algorithm to build statistical models of immune cell types. Images were also qualitatively assessed independently by a Pathologist as ‘high’, ‘moderate’ or ‘low’, for stromal and total immune cell content. Excellent agreement was found between manual assessment and total automated scores (*p* < 0.0001). Moreover, compared to single markers, a multi-marker classification of regulatory T cells (Tregs: CD3+/CD4+FOXP3+/PD1−) was significantly associated with disease-free survival (DFS) and overall survival (OS) (*p* = 0.049 and 0.032) of FOLFOX-treated patients. Our results also showed that PD1− Tregs rather than PD1+ Tregs were associated with improved survival. These findings were supported by results from an independent FOLFOX-treated cohort of 191 stage III CRC patients, where higher PD1− Tregs were associated with an increase overall survival (*p* = 0.015) for CD3+/CD4+/FOXP3+/PD1−. Overall, compared to single markers, multi-marker classification provided more accurate quantitation of immune cell types with stronger correlations with outcomes.

## Introduction

For early and locally advanced (stage I and II) colorectal cancer (CRC), the standard treatment of choice for low-risk patients is surgical resection. Subsequent oncological treatment decisions for non-metastatic CRC are based largely on the anatomical AJCC/UICC TNM staging classification^1^. After the MOSAIC study in 2004, patients with stage III CRC now commonly receive oxaliplatin/fluoropyrimidine/leucovorin (5-fluorouracil (5FU), FOLFOX; or xeloda/capecitabine, XELOX) as standard adjuvant treatment^2^. Of patients with stage III CRC treated with adjuvant chemotherapy, only ~20% will benefit from adjuvant FOLFOX, and 30% relapse within 2–3 years after surgery. Consequently, 80% of patients receive chemotherapy (and endure unnecessary toxicities) that yields no benefit^3^. However, improvements in the understanding of CRC heterogeneity are paving the way for more personalized approaches that combine both histological and molecular data for patient stratification and therapy selection, including selecting which patients will benefit from adjuvant chemotherapy^4,5^.

In the past decade, there has been an increasing interest in the impact of the tumor microenvironment (TME) on patient prognosis. Decreased risk of tumor progression and improved survival have been observed in solid tumors with high T-cell infiltration^6^. For CRC, the concept of an “Immunoscore” was introduced by Galon et al.; this evaluates CD3/CD8-positive immune infiltrates in the tumor core and tumor margin to classify “TNM-immune scores” for tumors^7^. In addition to Immunoscore, there have been numerous studies that reinforce the importance of tumor-infiltrating lymphocytes (TILs) as indicators of prognosis in CRC^8,9^. The importance of the immune contexture in CRC for patient prognosis logically suggests that immunotherapy could be a promising therapeutic approach^10^. Responsiveness to immunotherapy depends on several key factors, including high mutational loads (leading to high levels of tumor neoantigens), which are found in MMR-deficient (dMMR) microsatellite instability-high (MSI-high) CRC^11,12^. The immune checkpoint inhibitor (ICI) pembrolizumab has been approved by the US Food Drug Administration for patients with metastatic dMMR/MSI-high CRC. However, the majority of colorectal tumors (85–90%) are microsatellite stable (MSS), with low mutational burdens and exhibit no response to ICI therapy. Thus, chemotherapy remains the backbone therapy for MSS CRC.

With the unmet clinical need to better stratify stage III patients for possible adjuvant (or neo-adjuvant) chemotherapy and the opportunity to better quantify immune response using newer cell quantification methods, our goals were to: (1) compare multi-marker immune cell classification with immune cell scores determined by a Pathologist; and (2) investigate associations between single-marker versus multi-marker immune cell classification and patient outcomes.

## Results

### Pathologist scoring versus automated immune cell classification

The tissue microarray (TMA) cores from the patients were assessed by a Pathologist (M.B.L.) and, after exclusion criteria, 62 patients had 3 assessable cores, 99 had 2 assessable cores, whereas 7 patients had only 1 assessable core. Intra-tumor heterogeneity was reflected in intra-patient differences between the Pathologist’s immune and stroma scores. Specifically, from the 62 patients with 3 assessable cores, only 13 (19%) had the same immune score and 18 (29%) had the same stroma score for all three cores. For 5 (8%) patients, the immune score was different in each of the three cores, while for 6 (10%) patients, the stroma score was different in each of the three cores. This is to be expected given tumor histology variation in different core punches. From the 99 patients with two cores, 44 (44%) had the same immune score and 42 (42%) had the same stroma score in both tissue cores. In summary, for the 161 patients with more than one core, 104 (65%) showed immune heterogeneity and 101 (63%) showed stroma heterogeneity between their tissue cores. This highlights the inherent high degree of intra-tumor heterogeneity in CRC.

**Fig. 1:**
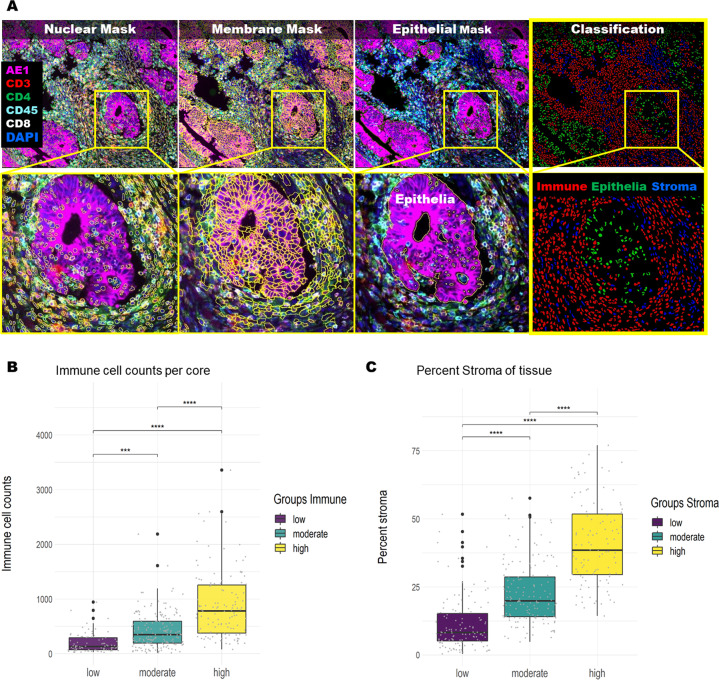
Comparison of tumor immune and stromal content evaluation by Cell DIVE and Pathologist. **A** Cell DIVE workflow: immunofluorescence staining with overlaying segmentation masks and resulting classification. Based on the classification data, an immune and stroma score was calculated per TMA core. **B** The immune score was calculated from the immune cell density as counts of segmented cells that were positive for any of the immune markers (CD45, CD3, CD4, CD8) in each core. The cores were grouped based on the Pathologist’s high-medium-low immune scores (*x*-axis) and each dot was the value of the Cell DIVE immune score (*y*-axis) per core. **C** The stroma score for each TMA core was calculated by counting segmented cells outside the epithelial mask that were negative for AE1 and immune markers and converting to ‘percent of total’ cells. The cores were grouped based on the Pathologist’s high-medium-low stroma scores (*x*-axis) and with each dot indicating stroma score (*y*-axis) per core. Statistical analysis was performed using Welch’s ANOVA and pairwise *t*-test (***P and ****P < 0.001 for all comparisons).

MBL performed visual inspection of the virtual Hematoxylin and Eosin (H&E) slides and assigned scores to each core of ‘high’, ‘moderate’ or ‘low’, for both stromal and immune cell content. We used the machine-learning workflow to create a quantitative cell classification-based immune and stroma score (Fig. 1A) to compare with the Pathologist’s scores. The Cell DIVE immune (*p* < 0.001; Fig. 1B) and stromal (*p* < 0.001; Fig. 1C) score values were significantly associated with the corresponding Pathologist’s scores. Therefore, the machine-learning-based Cell DIVE cell classification has potential to be used to evaluate tumor immune and stromal content.

### T-cell classification for single-marker and multi-marker (multiplexed) classification models

**Fig. 2:**
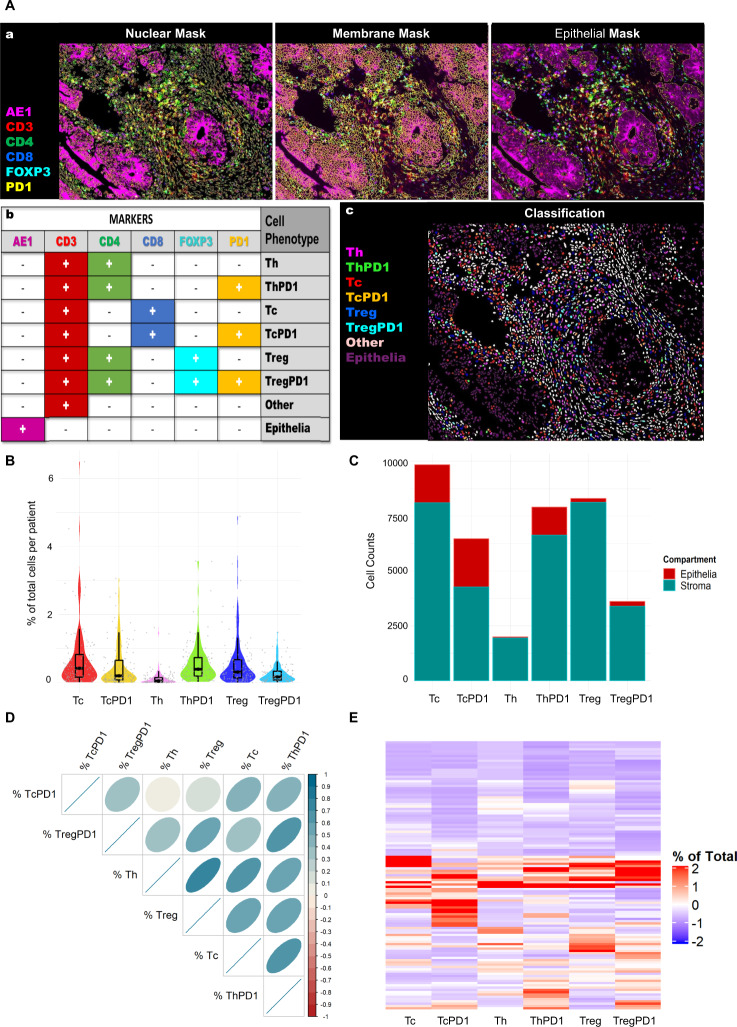
T-cell classification models. **A**(a) Representative multiplexed immunofluorescent tissue images and segmentation masks that were used for the multi-marker classification workflow. For single marker classification, the immune markers were assessed individually (CD3, CD4, CD8, FOXP3, PD1) and each cell was classified as positive or negative for each marker. **A**(b) Marker combination (AE1, CD3, CD4, CD8, FOXP3, PD1) was used for multi-marker classification workflow and based on the marker co-staining each cell was assigned a cell subtype as shown in the table. The eight multi-marker T-cell subtypes were as follows: T helper cells (Th), T helper PD1 (ThPD1), T cytotoxic (Tc), T cytotoxic PD1+ (TcPD1), T regulatory (Treg), T regulatory PD1 positive (TregPD1), Epithelial and other (non-lymphocyte, non-epithelial) cells. **A**(c) Illustration of the resulting multi-marker classification for the nuclear mask. **B** Distribution of calculated values for % of Total for each T-cell subtype, per patient. Each value represents the average of 2–3 assessable cores, per patient. **C** Total counts from all patients grouped as epithelial-associated cells (red) and stromal cells (green) for each multi-marker T-cell subtype. **D** Correlation matrix showing the relationship between different T-cell subtypes (Spearman’s correlation coefficients). A color-coded correlation scale is provided: blue ellipses represent positive correlations, while darker color and narrower ellipses correspond to larger correlation coefficient magnitudes. **E** Heatmap showing separation and clustering of patients based on % T cells of Total cells in tumor cores. Clusters based on the Ward.D agglomerative clustering method with Euclidean correlation distance measure.

In order to study the impact of different T-cell subtypes on patient prognosis in this adjuvant chemotherapy-treated cohort, we used a panel of T-cell biomarkers as described earlier. In addition, to single-marker analyses (CD3, CD4, CD8, FOXP3, PD1), multi-marker combinations were used to define subtypes (T cytotoxic (Tc), T cytotoxic PD1+ (TcPD1), T helper cells (Th), T helper PD1 (ThPD1), T regulatory (Treg), T regulatory PD1 positive (TregPD1), Fig. 2A). In the single-marker classification workflow, each one of these immune markers was analyzed individually, and each segmented cell was classified as either positive or negative for each marker. Since the individual markers were used to generate the multi-marker classification, it is not surprising that they were significantly correlated (*p* < 0.001; Supplementary Fig. 3). The demographic data of the patient cohort are summarized in Table 1.

**Table 1:** Demographic data of patient cohort.

	Overall
	(*N* = 117)
DFS (months)	
Mean (SD)	51.7 (27.9)
Median [Min, Max]	50.7 [2.40, 115]
IQR	[25.9 - 73.0]
OS (months)	
Mean (SD)	58.5 (24.7)
Median [Min, Max]	59.1 [9.20, 115]
IQR	[38.9–76.7]
sex	
Female	46 (39.3%)
Male	71 (60.7%)
T	
2	10 (8.5%)
3	70 (59.8%)
4	37 (31.6%)
N	
1	83 (70.9%)
2	34 (29.1%)
Age (years)	
Mean (SD)	59.2 (11.2)
Median [Min, Max]	61.0 [26.0, 79.0]
IQR	[52.0–67.0]
LNC	
Mean (SD)	21.3 (11.0)
Median [Min, Max]	19.0 [5.00, 73.0]
IQR	[14.0–25.0]
PLN	
Mean (SD)	3.05 (2.49)
Median [Min, Max]	2.00 [1.00, 13.0]
IQR	[1.00–4.00]
LVI	
No	49 (41.9%)
Yes	68 (58.1%)
Differentiation	
Moderate to well	99 (84.6%)
Poor	16 (13.7%)
Missing	2 (1.7%)
MSI status	
MSI	5 (4.3%)
MSS	30 (25.6%)
Missing	82 (70.1%)

*LNC* lymph node count, *PLN* positive lymph nodes, *LVI* lymphovascular invasion.

Representative immunofluorescent images of a single tissue core for the individual markers and the corresponding Segmentation Masks are illustrated in Supplementary Fig. 5. In the multi-marker classification workflow, all markers were assessed simultaneously (Fig. 2A(a)) and, depending on marker co-localization, segmented cells were assigned to the following classes (Fig. 2A(b/c)): PD1-negative T-helper (Th), PD1-positive Th (ThPD1), PD1-negative cytotoxic T cells (Tc), PD1-positive Tc (TcPD1), PD1-negative Treg and PD1-positive Treg (TregPD1).

To account for tumor heterogeneity, only patients with more than one core were used for the analysis (117 patients). Each T-cell subtype was calculated as a percentage of total cells per core, and the average percentage per patient was calculated. The distribution of T-cell subtypes across the cohort is shown in Fig. 2B; Tc and TcPD1 cells were the most abundant subtype associated with the epithelial compartment; however, overall, and as expected, the majority of each T-cell subtype was located in the stroma (Fig. 2C). All T-cell subtypes were generally positively correlated with each other, except that TcPD1 had minimal correlation with Th and Treg (Fig. 2D). Hierarchical clustering was used to assess the immune landscape of the patient cohort (Fig. 2E). Separation into two clusters, immune “hot” (higher immune cells) and “cold” (lower immune cells), showed that nearly 50% of patients were low in all T-cell subtypes; however, Kaplan–Meier analyses showed that their prognosis was similar to patients with higher level of T cells (Supplementary Fig. 6A). After separating into three clusters, the “immune-hot” cluster of patients with the highest infiltration of T-cell subtypes showed improved disease-free survival (DFS) and overall survival (OS) compared to the other two groups that had lower T-cell levels; however, this did not reach statistical significance (Supplementary Fig. 6B). Detailed summary statistics for T cells for the multi-marker classifications and single marker classifications are presented in Table 2.

**Table 2:** Summary statistics for multi-marker and single-marker subtypes.

	Overall
	(*N* = 117)
% Tc (of total cells)	
Mean (SD)	0.693 (0.925)
Median [Min, Max]	0.423 [0.0111, 6.49]
IQR	[0.156–0.826]
% TcPD1	
Mean (SD)	0.477 (0.619)
Median [Min, Max]	0.197 [0, 3.06]
IQR	[0.0798–0.652]
% Th	
Mean (SD)	0.133 (0.251)
Median [Min, Max]	0.0511 [0, 1.62]
IQR	[0.0103–0.144]
% ThPD1	
Mean (SD)	0.559 (0.593)
Median [Min, Max]	0.394 [0.0222, 3.57]
IQR	[0.189–0.729]
% Treg	
Mean (SD)	0.568 (0.732)
Median [Min, Max]	0.312 [0, 4.88]
IQR	[0.139–0.669]
% TregPD1	
Mean (SD)	0.262 (0.275)
Median [Min, Max]	0.166 [0.00471, 1.48]
IQR	[0.0723–0.331]
% CD3 (of total cells)	
Mean (SD)	3.18 (2.88)
Median [Min, Max]	2.52 [0.221, 22.3]
IQR	[1.36–4.31]
% CD4	
Mean (SD)	3.64 (3.52)
Median [Min, Max]	2.67 [0.126, 23.3]
IQR	[1.46–4.73]
% CD8	
Mean (SD)	2.17 (2.42)
Median [Min, Max]	1.35 [0.0283, 12.3]
IQR	[0.576–2.71]
% FOXP3	
Mean (SD)	1.58 (1.46)
Median [Min, Max]	1.19 [0.0114, 8.57]
IQR	[0.651–1.98]
% PD1	
Mean (SD)	0.669 (0.694)
Median [Min, Max]	0.395 [0.00943, 3.41]
IQR	[0.189–0.919]

**Fig. 3:**
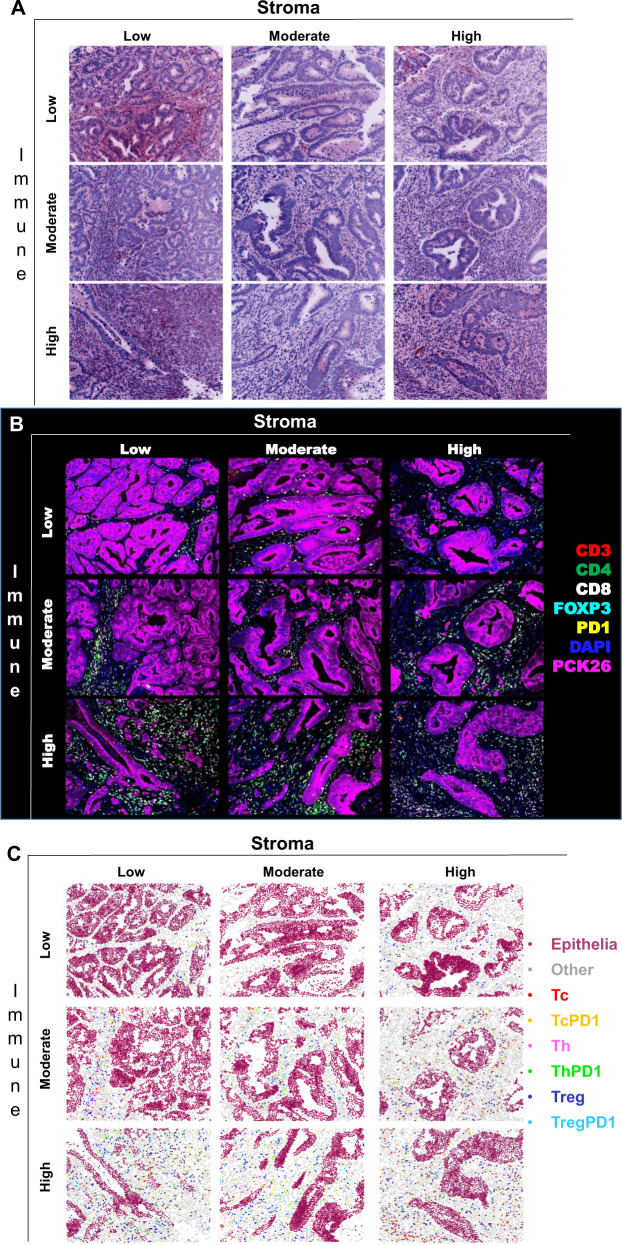
Representative images for each of the Pathologist’s immune (vertical) and stroma (horizontal) high, moderate and low scores. **A** Pseudo-colored virtual H&E (vH&E) images; **B** corresponding multiplexed immunofluorescence images for all classification markers; **C** tissue mappings after classification with cells color-coded for each cell subtype. All panels illustrate the same images. The top left image in each panel is a representative immune-Low, stroma-Low tissue core while the bottom right image is a representative immune-High, stroma-High tissue core.

In Fig. 3 representative images of virtual H&Es, immunofluorescent images and tissue mappings with color-coded cell classifications are illustrated. The selected images are representative of all 9 Stroma-Score/Immune-Score combinations from the Pathologist review. This shows that multiplexing can be used to identify multiple subtypes of immune cells simultaneously, allowing for associations and potential cross-talk between distinct cell subtypes in the TME to be assessed.

### T-cell infiltration and patient prognosis

As proof of concept for the applicability of this approach for identification of prognostic immune biomarkers, we next determined the prognostic value of the single and multiplexed markers in this FOLFOX-treated stage III patient cohort. The correlation of each T cell type with clinical endpoints (DFS and OS) was analyzed using univariate and multivariate Cox proportional hazards models and Kaplan–Meier analyses. In this analysis, we used the average percentage of T cells for each patient (average of each patient’s cores).

**Fig. 4:**
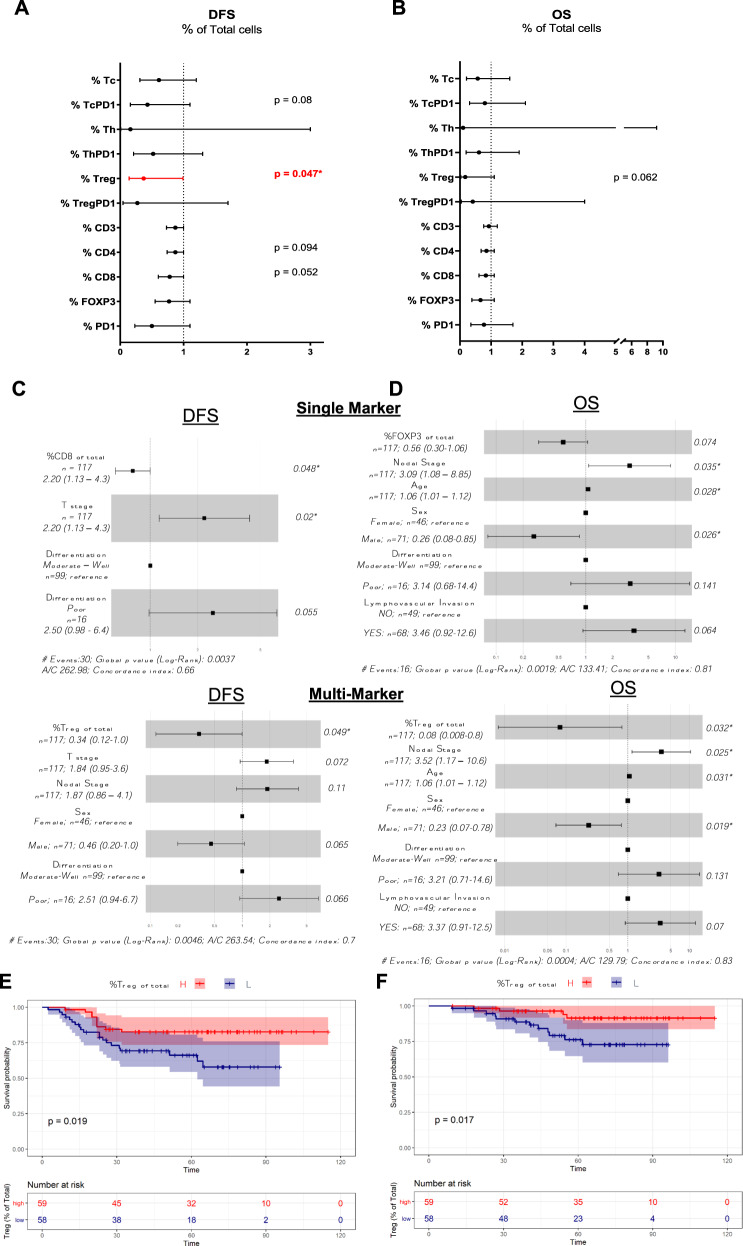
Assessment of DFS and OS using the average percentage of T cells for each patient (average of each patient’s cores). Forest plots for multi-marker classification (Tc, TcPD1, Th, ThPD1, Treg, TregPD1) and single-marker classification (CD3, CD4, CD8, FOXP3, PD1) were generated for DFS (**A**) and OS (**B**). HRs, 95% CIs, and *p* values from likelihood ratio tests from univariate Cox proportional hazards models were calculated. In the multivariate analysis, the biomarkers were adjusted for clinical variables (T, N, age, sex, nodal count, positive nodes, differentiation, lymphovascular invasion) for DFS (**C**) and OS (**D**). Kaplan–Meier curves demonstrating univariate survival analysis for average Treg scores dichotomized on the median for DFS (**E**) and OS (**F**). Differences in Kaplan–Meier survival curves are presented as log-rank *p* value.

In the univariate analyses, the Forest plots in Fig. 4 demonstrate that none of the single immune markers was significantly associated with DFS (Fig. 4A) or OS (Fig. 4B), whereas the level of Treg cells (CD3+/CD4+/FOXP3+/PD1−) from the multi-marker machine-learning classification was significantly associated with longer DFS (HR = 0.37, 95% CI = 0.14–0.99, *p* = 0.047). For the multivariate analysis, the model initially included the clinical variables: T, N, age, sex, nodal count, positive nodes, differentiation and lymphovascular invasion together with single- and multi-marker immune scores. Backward elimination was used to select variables for the final model. For DFS in the single-marker model, CD8 remained in the final model and was positively associated with longer DFS (multivariate adjusted HR = 0.78, 95% CI = 0.6–1.0, *p* = 0.048; Fig. 4C) and, in the multi-marker model, Tregs remained positively associated with longer DFS (multivariate adjusted HR = 0.34, 95% CI = 0.12 - 1.0, *p* = 0.049; Fig. 4C). For OS in the single-marker model, FOXP3 remained in the final model but did not reach significance (multivariate adjusted HR = 0.56, 95% CI = 0.297–1.06, *p* = 0.074; Fig. 4D) and in the multi-marker model Tregs remained positively associated with longer OS (multivariate adjusted HR = 0.08, 95% CI = 0.0079–0.8, *p* = 0.032; Fig. 4D). The detailed Forest plots for the multivariate models for clinical variables only are shown in Supplementary Fig. 7.

In order to facilitate comparison with previously published results, Treg levels were divided into high and low groups using the sample median as the cut-off, and Kaplan–Meier analyses were performed for curves for DFS and OS (Fig. 4E, F). Similar to the univariate and multivariate analyses above, Treg*-high* patients had improved DFS (*p* = 0.019) and OS (*p* = 0.017) than Treg-low patients. Kaplan–Meier curves for all single-marker and multi-marker classes dichotomized on the median are included in Supplementary Fig. 8. Sub-regional analysis based on the percentage of immune cell subtypes located in the stroma or located within/associated with the epithelial compartment and association with outcome are shown in Supplementary Table 3.

**Fig. 5:**
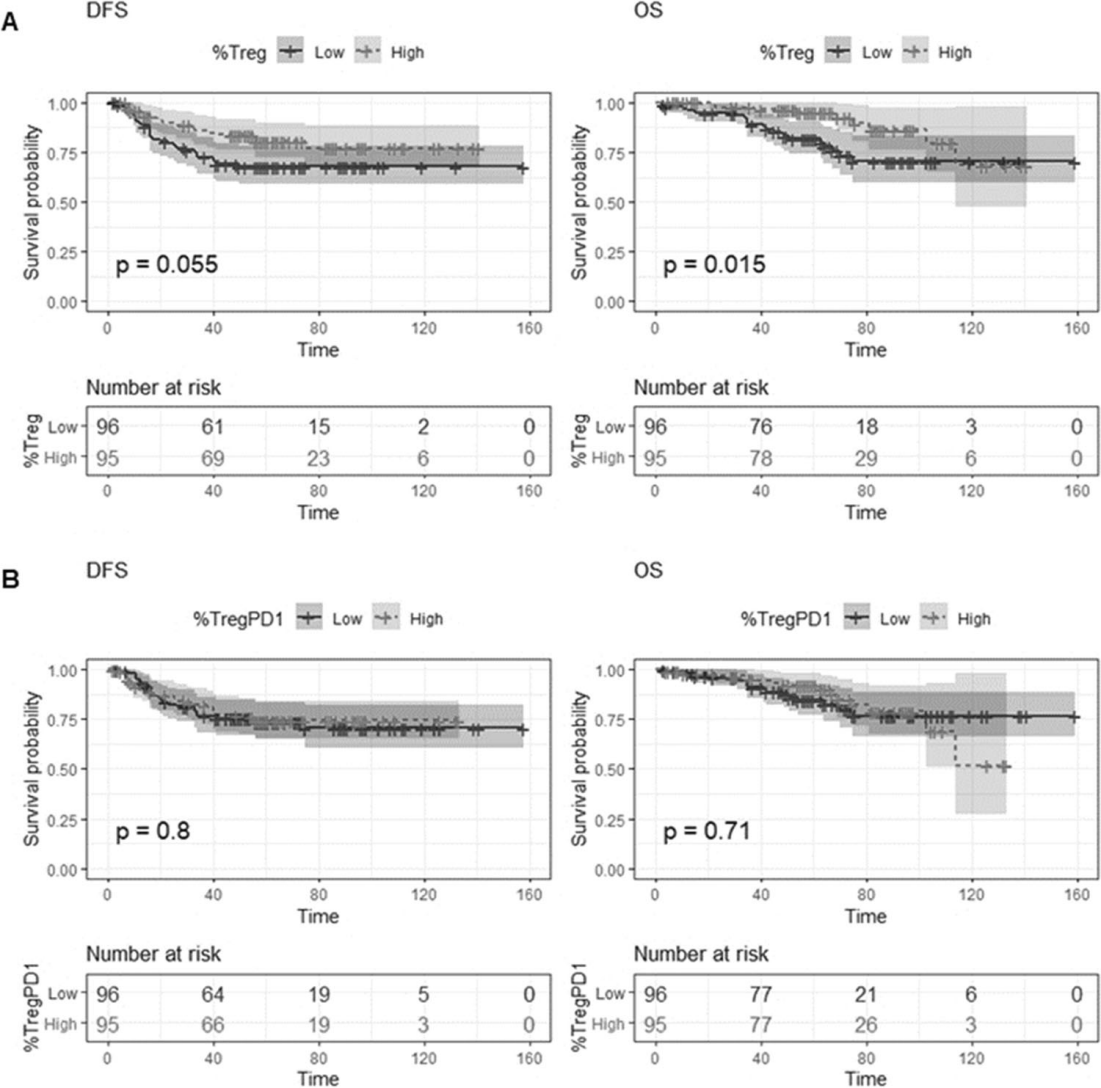
Validation cohort. Kaplan–Meier curves demonstrating univariate survival analysis for average Treg (**A**) and TregPD1 (**B**) scores dichotomized on the median for DFS and OS. Differences in Kaplan–Meier survival curves are presented as log-rank *p* value.

Importantly, similar results were obtained in an independent FOLFOX-treated stage III patient cohort, where Treg-high (CD3+/CD4+/FOXP3+/PD1− cells) patients had improved DFS (Fig. 5A), although this just failed to reach significance (HR = 0.56, 95% CI = 0.31–1.02, *p* = 0.057), and significantly improved OS (Fig. 5A) (HR = 0.4, 95% CI = 0.18–0.85, *p* = 0.02). In further agreement with the discovery cohort, the Treg-PD1+ cells were not associated with DFS or OS (Fig. 5B).

### T-cell infiltration and patient prognosis for immune hot-spot

**Fig. 6:**
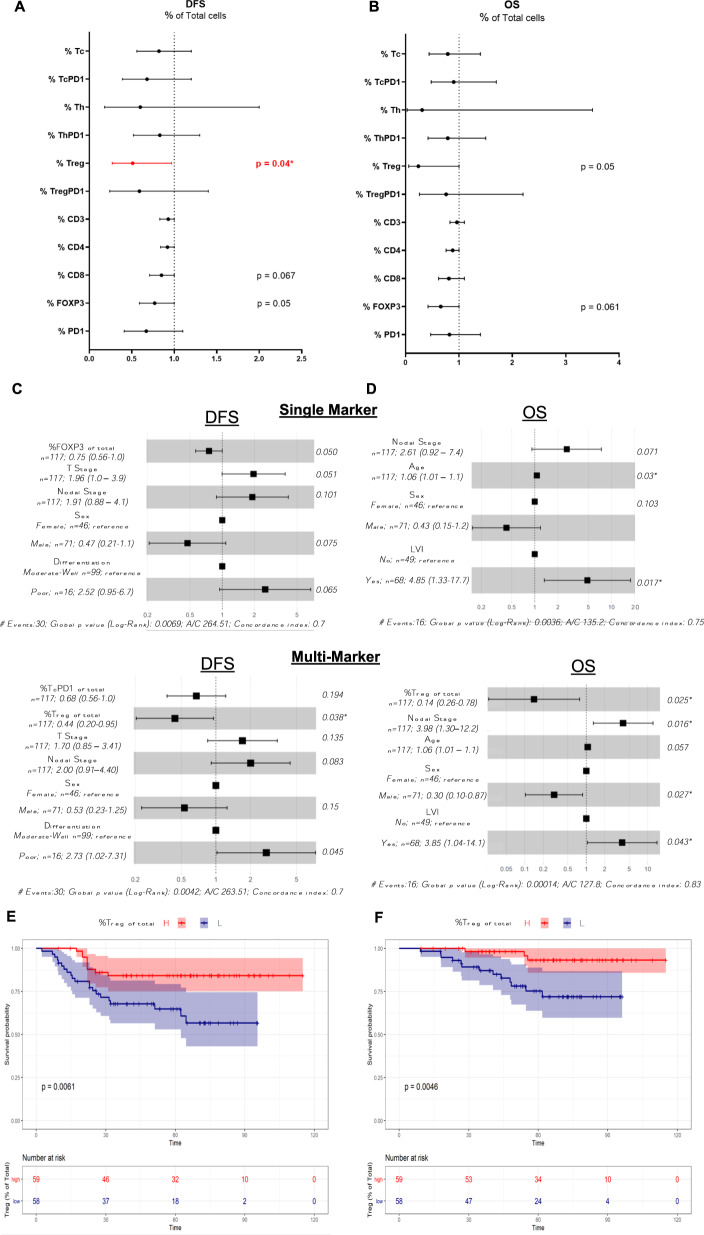
Survival estimates for DFS and OS for T-cell scores in immune “hot-spot” regions. Forest plots for multi-marker classification (Tc, TcPD1, Th, ThPD1, Treg, TregPD1) and single-marker classification (CD3, CD4, CD8, FOXP3, PD1) were generated for DFS (**A**) and OS (**B**). HRs, 95% CIs, and *p* values from likelihood ratio tests from univariate Cox proportional hazards models were calculated. In the multivariate analysis, the biomarkers were adjusted for clinical variables (T, N, age, sex, nodal count, positive nodes, differentiation, lymphovascular invasion) for DFS (**C**) and OS (**D**). Kaplan–Meier curves demonstrating univariate survival analysis for Treg levels in the immune hot-spot regions dichotomized on the median for DFS (**E**) and OS (**F**). Differences in Kaplan–Meier survival curves are presented as log-rank *p* value.

In order to account for tumor immune heterogeneity, the average percentage of T cells in multiple cores was used for the above data analyses. However, this could dilute the impact of very high but very localized immune cell infiltrates. We hypothesized that by focusing our analyses on the available cores with the *highest* tumor immune regions, we might uncover additional prognostic information; therefore, we repeated the above analyses for the one core per patient with maximum T-cell density for each subtype. Cox proportional hazards regression analysis and Kaplan–Meier plots were performed as above. In the univariate analysis, none of the single markers was significantly associated with survival. For the multi-marker classification Treg levels were significantly associated with DFS (HR = 0.51, 95% CI = 0.27–0.97, *p* = 0.04; Fig. 6A) and were borderline significant for OS (HR = 0.24, 95% CI = 0.059–1, *p* = 0.05; Fig. 6B).

In the multivariate analysis, for DFS in the single-marker model, FOXP3 remained in the final model (multivariate adjusted HR = 0.75, 95% CI = 0.56–1.0, *p* = 0.05) and had borderline statistical significance (Fig. 6C), and, in the multi-marker model, Treg and TcPD1 remained in the final model and Treg remained statistically significant (for TcPD1: multivariate adjusted HR = 0.68, 95% CI = 0.38–1.22, *p* = 0.194; for Treg: multivariate adjusted HR = 0.44, 95% CI = 0.20–0.95, *p* = 0.038). For OS, none of the single markers remained in the final model. In the multi-marker model, Treg levels remained in the final model and were significantly associated with improved OS (multivariate adjusted HR = 0.14, 95% CI = 0.026–0.78, *p* = 0.025) (Fig. 6D).

As previously, Kaplan–Meier curves for all single-marker and multi-marker classes dichotomized on the median were generated. Again, high levels of PD1-negative Tregs were significantly associated with better prognosis: DFS (*p* = 0.0061); and OS (*p* = 0.0046) (Fig. 6E, F). In this “hot-spot” analysis, high CD4 levels also correlated with better prognosis but with borderline significance, while no other single or multiplex marker had prognostic significance (Supplementary Fig. 9). Sub-regional analysis based on the percentage of immune cell subtypes located in the stroma or located within/associated with the epithelial compartment and association with outcome are shown in Supplementary Table 4.

## Discussion

A large number of multigene signatures using tumor gene expression profiles have emerged in the last decade, such as Consensus Molecular Subgroups (CMS) and CRC Intrinsic Subtypes (CRIS), which classify patients into molecular subtypes for risk prediction^19–21^. However, this approach is therapeutically valuable only under the assumption that highest-risk patients will also be the most responsive to chemotherapy. This is not the case and, in fact, CMS4 patients who are predicted to have poor prognosis do not benefit from intensive adjuvant chemotherapy^22^. We recently reported that stage II patients with CMS2/CRIS-C tumors, which demonstrate low levels of CD8-positive TILs benefit from adjuvant chemotherapy. In stage III patients, benefit from chemotherapy was particularly apparent in CMS2/CRIS-C and CMS2/CRIS-D patients^5^. However, transcriptional profiling is not routinely available or applied in clinical practice. Ideally, a clinical test to triage patients for adjuvant chemotherapy that could be performed rapidly on a single formalin-fixed paraffin-embedded (FFPE) tumor section would be extremely useful.

Over the last decade, there has been a growing body of evidence that multiplexed imaging methods and spatial cell analysis, including immunofluorescence-based^23–26^, mass cytometry^27,28^, multiplexed ion beam imaging by time-of-flight (MIBI-TOF)^29^ and spatial transcriptomics^30^, can provide critical new insights into spatial relationships between tumor and immune cells, as well as characterization of the TME^31–35^. Since multiplexed imaging allows multiple markers to be stained and quantified simultaneously in a single tissue section, this avoids potentially confounding cellularity changes that are introduced by sequential sectioning, thereby opening up the potential to develop accurate multi-marker classifications. Here, we used multiplexed immunofluorescent imaging to compare the prognostic potential of single marker and multiplex analyses of markers associated with helper, cytotoxic and regulatory T cells in a single FFPE section. To evaluate the real-World potential of the methodology, we initially determined how evaluation of immune and stroma burden compared to immune and stroma scoring by a gastrointestinal Pathologist. Our machine-learning cell classification method showed significant correlation with the Pathologist’s assessment, supporting the potential clinical utility of the platform.

Using a combination of ten markers for cell classification, we went on to show that we could quantify six sub-classes of T cells using a single TMA section. Our results showed that high levels of CD3+/CD4+/FOXP3+/PD1− Treg cells were associated with better DFS in this FOLFOX-treated cohort. These results were supported by analysis of an independent stage III FOLFOX-treated cohort. We also assessed the association between different T-cell subpopulations and disease outcome using the core with the highest T-cell infiltration (or the “immune hot-spot” core). We reasoned that, while using the core average accounts for heterogeneity and may be more representative of an entire tumor section, the immune hot-spot core could be more indicative of how likely patients were to relapse by more accurately reflecting the extent of anti-tumor immunity. However, comparing the two workflows, the results were similar, especially in the univariate analysis, where none of the single markers was significant, while Treg/PD1-negative cells were significantly associated with DFS in both workflows. In the multivariate analysis, the results were also comparable for the multi-marker classes, with Treg/PD1-negative cells remaining significant.

Tregs regulate the activity of multiple immune cells, such as CD4+ and CD8+ effector cells, macrophages and dendritic cells^36^. In apparent contrast to our findings, high Treg levels have been associated with poor clinical outcomes in different cancers, including CRC^37–39^. However, in agreement with our study, others have found that high Treg levels associate with better prognosis in CRC patients^40–44^. There are a number of reasons that could be responsible for these apparently contradictory results. For example, differences in the study cohorts, such as stage and whether patients were treated with chemotherapy, in addition to technical differences in detection and variable thresholds for scoring^45^. Importantly, the conflicting results may be due to the use of single biomarkers that fail to reflect the Treg versatility and plasticity. FOXP3 is routinely used as a Treg biomarker in clinical studies. However, it has limitations since it is not exclusively expressed by Treg cells. For example, FOXP3 can also be expressed in dividing, activated T effector cells^46,47^. In addition to FOXP3, some Treg subtypes express other molecules that increase their immunosuppressive capacity, and these highly suppressive Treg cells have been detected in CRC patients^48–51^. The immunosuppressive activity of PD1 has made it and its ligand PD-L1 key targets for immune oncology. Our results show that it is PD1-negative Tregs rather than PD1+ Tregs that are associated with improved prognosis in two independent cohorts. The enrichment of PD1-negative Tregs may reflect the presence of an active inflammatory response rather than the establishment of an immunosuppressive TME; this would explain the association which we observed with improved prognosis in this chemotherapy-treated stage III cohort. Therefore, relying solely on FOXP3 as a marker of Tregs may be the cause of some of the inconsistencies in the literature regarding Treg and CRC prognosis. The inter-relationships between immune cell lineages and spatial heterogeneity of the tumor are also of critical importance for understanding how tumors progress and for evaluating therapy options. For example, the role of the TME and epithelial and stromal domains and their contribution to tumor progress was demonstrated by Uttam et al.^34^ who used multiplexed imaging and cell analysis of 55 biomarkers (using the same platform as this study) in 432 stage II chemo-naive CRC patients. Their spatial analytics computational and systems biology platform (SpAn) showed the prognostic significance of spatial domains and networks within the tumor^34^. Combining this type of spatial analysis with immune cell phenotypes will provide powerful new insights into tumor progression and therapy options in CRC patients.

The limitations for adoption of this methodology in the clinic would include the additional cost for the automated fluorescent imaging platform. Most importantly, as this powerful analytical tool produces large amount of multidimensional data, user-friendly machine-learning methodologies and analytical workflows would need to be customized. One technical limitation of our study is the use of TMA cores instead of whole tissue slides (WTS). TMAs have multiple advantages compared to WTS, such as prevention of batch effects, minimizing of analysis times and costs, and preservation of valuable biomaterials. While non-perfect correlations between TMAs and WTS have been reported, analysis of WTS is more expensive, time-consuming and generates even more data, with subsequent issues for data storage interpretation.

In summary, we show that multiplexed analyses can be used to accurately identify and enumerate subpopulations of T cells. We also provide evidence that compared to single marker (FOXP3) assessment of Tregs, a multi-marker classification (CD3+/CD4+/FOXP3+/PD1−) has superior clinical potential to identify patients who have a better prognosis following adjuvant FOLFOX treatment. Overall, we conclude that automated multi-marker immune cell classification provides accurate quantification of immune cell subtypes and has real-world potential for evaluation of prognostic biomarkers.

## Materials and methods

### Patient cohorts

Five TMAs from FFPE tissue blocks with up to three 1-mm-diameter cores per patient were prepared from 170 patients with stage III CRC. The punches were taken from the center of the tumor based on identification by a Pathologist (Prof Manuel Salto-Tellez, Queen’s University Belfast) and the invasive front was not included. The patient samples were collected from three Research Centres: Beaumont Hospital (RCSI Hospital Group, Ireland), Queen’s University Belfast (UK), and Paris Descartes University (France), and the TMAs were constructed at Queen’s University Belfast. The TMAs from Ireland and France had three cores from each tumor and the TMAs from UK had two cores from each tumor. The TMA design is shown in Supplementary Fig. 1. The pathological stage was determined by the AJCC 7th edition TNM staging system. All Centers provided ethical approval for this study and informed consent was obtained from all participants (NIB12-0034). This was a retrospective study, and the patients were recruited during 2005–2012. None of the patients had received any sort of ICI therapy prior to resection. At the patient level, the exclusion criteria based on tissue block or clinical data were as follows: (i) poor tissue quality or no tumor cells in tissue; (ii) loss of follow-up or recurrence and/or death within less than two months from surgical resection; (iii) absence of chemotherapy treatment; (iv) positive resection margins; (v) tumor site was appendix; (vi) stage II or IV disease; (vii) only one assessable core remaining after applying all exclusion criteria. At the tissue core level, individual cores on the TMA were excluded for assessment after pathology TMA slide review if no or minimal viable tumor was present for evaluation (i.e. minimal or no tumor tissue, heavily artefacted tissue, extensive tumor necrosis, extensive presence of normal adjacent tissue). After applying exclusion criteria from the original patient cohort, the remaining training data comprised 117 stage III patients, who were all treated with 5FU-based adjuvant chemotherapy (predominantly FOLFOX or XELOX).

### Validation cohort

Eleven TMAs from FFPE tissue blocks with two 1-mm-diameter TCs per patient were prepared from 388 patients with stage II and III CRC (*n* = 287 stage III patients). The punches were taken from the center of the tumor based on identification by a Pathologist (J.S., Memorial Sloan Kettering Cancer Center) and two adjacent normal cores were also included for each patient. However, for the purpose of validation, the data set was filtered to only include TCs and patients receiving FOLFOX treatment. Clinical details are included in Supplementary Table 1.

### Multiplexed immunofluorescence analysis of TMAs

Multiplexed immunofluorescence staining of the CRC TMAs was performed as previously described^13^ using Cell DIVE™ (formerly GEHC, now part of Leica Microsystems, Issaquah, WA), a multiplexed immunofluorescence microscopy method allowing for multiple protein markers to be imaged and quantified at cell level in a single tissue section. Briefly, FFPE tissue slides were de-paraffinized and rehydrated, underwent a two-step antigen retrieval, and were then stained for 1 h at room temperature using a Leica Bond autostainer. All antibodies were characterized per the previously described protocol^13^ and when possible, antibodies in routine clinical use were employed. After downselection, each antibody was conjugated with either Cy3 or Cy5 bis-NHS-ester dyes using standard protocols as previously described^13^. The entire core underwent multiplexed immunofluorescence staining and imaging for a total of 24 markers listed in Supplementary Table 2. The markers of interest for this study included CD3, CD4, CD8, FOXP3, CD45, NaKATPase, S6, pan-cytokeratin and AE1 and DAPI nuclear stain. All samples underwent DAPI imaging in every round, and background (inherent tissue autofluorescence prior to staining) imaging for the first five rounds and every three rounds thereafter.

### Image processing, single-cell segmentation

Using Cell DIVE automated image pre-processing software, all images were registered to baseline using DAPI and underwent background autofluorescence subtraction, illumination and distortion correction. DAPI and Cy3 autofluorescence images were used to generate a pseudo-colored image, which visually resembles a H&E stained image, which we refer to as a virtual H&E (vH&E). This visualization format helps tissue quality control (QC) review and facilitated review of tumor morphology and lymphocytes. All cells in the epithelial and stromal compartments were segmented using DAPI and pan-cytokeratin, while S6, and NaKATPase were used for subcellular analysis of epithelial cells. Each segmented cell was assigned an individual ID and spatial coordinate, as previously described^13–16^. Post segmentation, several QC steps were conducted (described in detail in Berens et al.^17^), including visual review and manual scoring of tissue quality and segmentation for every image, and the CONSORT flow diagram with exclusion criteria is summarized in Supplementary Fig. 1. Briefly, each image was reviewed for completeness and accuracy of segmentation masks in each subcellular compartment and tumor and stroma separation. Average biomarker intensity was calculated for each cell and the following additional cell filtering criteria were applied: (1) epithelial cells were required to have either 1–2 nuclei; (2) each subcellular compartment (nucleus, membrane, cytoplasm) area had to have >10 pixels and <1500 pixels; (3) cells had to have excellent alignment with the first round of staining (round 0); (4) cells were at >25 pixels distance from the image margins; (5) cell area for nuclear segmentation mask was >100 or <3000 pixels, (6) duplicates.

### Immune cell annotation workflow for cell classification—FOLFOX cohort

For each of the single-marker models, the cell classification models were separately trained for each individual marker. For each model, two classes of cells were annotated: marker positive (CD3+, CD4+, CD8+, FOXP3+, and PD1+) or negative cells. In total, five models were generated for the five individual markers. The multi-marker cell classification model involved using all relevant cell phenotype markers simultaneously, in one single model. The table in Fig. 2A(ii) shows the combinations of markers that determined the eight immune cell classes. All trained models are linear-kernel support vector machines (SVMs). The features included in the model were the mean and standard deviation of the marker’s intensity expression within identified cells. A minimum of five images were used for the model training (one core from each TMA slide with the max mean intensity of the marker of interest) and approximately ten cells per marker per image were annotated (roughly 10 cells per core and up to 50 cells for each class) in the first training. As shown in Supplementary Fig. 2, the annotated process is aided by the use of the cell segmentation masks, which reduces the risk of false positives from artefacts etc. Training accuracy (i.e., the number of training annotations that were correctly predicted) was between 69 and 100% for single markers and 78% for the multi-marker model. Training error is generated from an SVM that was trained on the entire dataset, to address the low relative presence of some markers. This was the model used for classification predictions of all the cells. Further, the images and predictions were visually evaluated, and the model was re-run with additional annotations. The initial annotations were conducted on cores with high numbers of immune cells, followed by intermediate and lower numbers of immune cells. Test accuracy was 70% ± 15% for single marker models and 44% for the multi-marker model. The test accuracy numbers were obtained by training SVM models with threefold cross validation. We attribute the lower accuracy of the multi-marker model to the larger number of classes (8) and the relatively small, unbalanced sample size once the data is split into 3 groups. We conducted a further extensive visual verification of the cell types vs predictions and correlated the counts of the multi-marker classification vs each individual cell types, shown in Supplementary Fig. 3. Further, there was good agreement between the FOXP3 counts and Treg counts in each core (Supplementary Fig. 4), with the Treg counts (CD3+CD4+FOXP3+and PD1±) generally lower than the total FOXP3 counts (as expected).

### Automated immune cell classification workflow for validation cohort

For the validation cohort, a modification to the immune cell classification workflow was used whereby a larger training set for CD3+, CD20+, CD4+, CD8+ FOXP3+, and PD1+ cells was automatically generated. This method was recently reported by Santamaria-Pang et al.^15^. The advantage of this method over the earlier version is that many more annotations can be automatically generated vs the earlier version, which relies on intensive annotations over multiple iterations to improve model performance. Briefly, the autofluorescence-removed images were segmented at cellular level to identify cells that were potentially positive for each marker via intensity and morphological criteria. These candidate annotations are then correlated with segmented nuclei and potential annotations with no corresponding nucleus are discarded. The remaining annotations are now the automatically generated training set. In the second step, a probability model is inferred from the automated training set. The probabilistic model captures staining patterns in mutually exclusive cell types and builds a single probability model for each marker. Manual annotations of the cell types (using a similar workflow shown in Supplementary Fig. 2) were also used to validate the algorithm performance with accuracy levels ranging from 70–100% for predicted vs annotated cells (150–500 cells annotated per marker, depending on abundance). After cell-level predictions were made for each marker, they were combined to generate multi-marker immune cell classification for each cell, including cytotoxic, helper, and r;egulatory T cells ± PD1. The manual and automated approaches were compared in independent CRC dataset and showed excellent correlations (correlations were >0.90 for single markers and >0.80 for multi-marker classifications).

### Pathologist scoring

Gastrointestinal Pathologist (M.B.L.) performed visual inspection of the virtual H&E slides generated from the DAPI and autofluorescence images^13,18^ for the 419 TMA cores from the FOLFOX study. After applying exclusion criteria described earlier, 28 cores were excluded and 391 cores were assessed. MBL assigned two qualitative scores to each core comprising either ‘high’, ‘moderate’ or ‘low’ scores, one for stromal cell content and one for immune cell content. For stroma, a high score was assigned when the stromal area was higher than the epithelial area; a moderate score was assigned when the stromal and the epithelial areas were equivalent; and a low score was assigned when the stromal area was lower than the epithelial area. The immune score was based on lymphoid cell abundance in the tissue core. For equivalent comparison of the Pathologist stroma and immune score with Cell DIVE automated scores the following steps were taken: (1) “Stromal cells” were defined as DAPI positive cells that were negative for all markers and outside the epithelial segmentation mask. The stroma score was calculated as the percentage of non-immune stromal cells in all segmented cells in the non-epithelial region. (2) “Immune cells” were defined as segmented cells that were positive for any of the immune markers (CD45, CD3, CD4, CD8) and negative for the AE1 epithelial marker. The immune scores were calculated from the counts of all segmented immune cells. (3) “Epithelial cells” were defined as segmented cells that were positive for AE1 staining and were within the Epithelial Segmentation Mask^15^.

### Statistical analysis

For comparison of quantitative stroma and immune scores with the Pathologist scores, the scores were categorized based on the Pathologist’s three qualitative groups (high–moderate–low). Statistical analysis for comparison of group means was performed using Welch’s ANOVA and pairwise *t*-test. The association of the single-marker and multi-marker classified immune cells with clinical outcome was evaluated using both univariate and multivariate analyses with adjustment for clinico-pathological confounders (T, N, age, sex, nodal count, positive nodes, lymphovascular invasion, differentiation) in the multivariate Cox proportional hazards models. For the final multivariate models, the variables were subjected to backward elimination and the variables that did not contribute to model fit were removed. The final multivariate model was tested for multi-collinearity and proportional Hazards assumption. Variables with variance inflation factor >2 were removed, and the remaining variables were re-subjected to backward elimination. The relative quality and goodness of fit of models was examined using Harrell’s C-index, and the model choice was determined by the Akaike Information Criterion. The T-cell subtypes were counted and analyzed as continuous variables after being transformed to ‘Percent of total’ tissue segmented cells, per patient. When the patients had multiple cores, the average percent of the assessable cores was calculated. For the immune hot-spot, we calculated the total counts of T cells in each core (CD3 counts for single markers and sum of all T cell subtypes for the multiplexed model). From the 117 patients, the cores with the highest number of CD3 or T cells (immune hot-spot core) were selected for further analysis. For survival analyses, the T cell subtypes calculated as % of total tissue cells were dichotomized at the median, and the Kaplan-Meier method was used to plot survival curves with the log-rank test used for comparisons. No adjustments were made for multiple comparisons. Hypothesis testing was performed at the 5% significance level. The endpoints studied were DFS and OS. DFS was the time between the study entry and either the date of the first recurrence, or the date that the last follow-up took place. OS was the time between the date of study entry and either the date of death from any cause, or the date of the last follow-up. All statistical analyses were performed in R Version 3.5.1 (https://cran.r-project.org).

## Supplementary information

Supplementary Figures

## Data Availability

Please contact the corresponding author for further information/access to data. Supplementary information is available at Modern Pathology’s website.
