# Supplementary material for: Stratification of chemotherapy-treated stage III colorectal cancer patients using multiplexed imaging and single-cell analysis of T-cell populations
Source: Mod Pathol. 2021 Nov 3;35(4):564–76. doi: 10.1038/s41379-021-00953-0 (PMC8964416; doi:10.1038/s41379-021-00953-0)
Supplement: Supplementary file 1 — Supplementary Figures [file 41379_2021_953_MOESM1_ESM.pdf]

Supp.Table 1: Demographic data of validation cohort

	**Overall (N=191)**
**DFS (months)**	
Mean (SD)	54.6 (33.0)
Median [Min, Max]	57.0 [2.00, 158]
**OS (months)**	
Mean (SD)	63.0 (30.5)
Median [Min, Max]	61.0 [2.00, 159]
**sex**	
female	101 (52.9%)
male	90 (47.1%)
**T**	
1	7 (3.7%)
2	22 (11.5%)
3	128 (67.0%)
4	34 (17.8%)
**N**	
1	129 (67.5%)
2	62 (32.5%)
**age (years)**	
Mean (SD)	59.3 (12.6)
Median [Min, Max]	59.0 [28.0, 88.0]
**LNC**	
Mean (SD)	21.3 (11.0)
Median [Min, Max]	19.0 [5.00, 73.0]
**PLN**	
Mean (SD)	3.28 (2.89)
Median [Min, Max]	2.00 [0, 14.0]
**LVI**	
no	48 (25.1%)
suspected	15 (7.9%)
yes	128 (67.0%)
**differentiation**	
moderate to well	147 (77.0%)
poor	44 (23.0%)
**MSI status**	
MSI	16 (8.4%)
MSS	175 (91.6%)

*LNC=Lymph Node Count, *PLN=Positive Lymph Nodes, *LVI=Lymphovascular Invasion

Supplementary Table 2: Antibodies, clones and conjugates used in this study.

Target	Clone	Vendor	Catalog	Concentratio	Staining round
APAF-1	2E12	Millipore	MAB3053	5	1
Bak	D4E4	Cell Signaling	12105	5	5
Bax	E63	Abcam	ab216985	10	4
BCL-2	124	Lifespan	LS-C389442	5	1
Bcl-xL	7D9	Thermo	MS-1334	10	6
CA9	polyclonal	Thermo	PA1-16592	15	11
Caspase-3 (Pro+cleaved)	D3R6Y	Cell Signaling	14214	10	3
Caspase-9	96.1.23	Santa Cruz	sc-56076 A647	5	2
**CD3**	F7.2.38	Dako	M7254	10	12
**CD4**	EPR6855	Abcam	ab181724	5	9
**CD8**	C8/144B	Dako	M7103	5	7
**CD45**	2B11 + PD7/26	Dako	M0701	10	10
**Cytokeratin AE1**	AE1	eBioscience	14-9001	2.5	8
**Cytokeratin PCK26**	PCK26	Sigma	C1801	5	7
**FOXP3**	206D	Biolegend	320014	10	8
Glut-1	EPR3915	Abcam	ab196357	5	11
HLA I	EMR8 5	Abcam	ab70328	10	10
Ki67	SP6	Zeta	Z2031	10	9
MCL-1	Y37	Abcam	ab186822	10	2
**NAKATPase**	EP1845Y	Abcam	ab167390	5	6
**S6**	C-8	Santa Cruz	sc-74459 A647	5	13
Smac	79-1-83	Cell Signaling	2954	10	4
**PD1**	EPR4877(2)	Abcam	ab201825	5	12
XIAP (API3)	polyclonal	Thermo	APH937	7.5	5

Supplementary Table 3: Hazard Ratios for single-marker and multi-marker T cell subsets for average of cores.

Multi-Marker

% of Total	DFS			OS		
**Stroma**	**HR**	**95% CI for HR**	**p.value**	**HR**	**95% CI for HR**	**p.value**
**Tc**	0.62	0.3-1.3	0.2	0.62	0.22-1.7	0.36
**TcPD1**	0.4	0.11-1.4	0.15	0.92	0.29-3	0.89
**Th**	0.13	0.0055-3	0.2	0.1	0.0011-9.8	0.33
**ThPD1**	0.51	0.18-1.4	0.19	0.65	0.2-2.2	0.48
**Treg**	0.38	0.14-1	0.052	0.17	0.025-1.1	0.067
**TregPD1**	0.3	0.05-1.8	0.19	0.49	0.055-4.3	0.52
**Epithelia-associated**						
**Tc**	0.097	0.0034-2.8	0.17	0.024	7.6e-05-7.5	0.2
**TcPD1**	0.066	0.0035-1.2	0.07	0.19	0.007-5.2	0.33
**Th**	3.90E+07	0.0026-6e+17	0.14	7.40E-12	3e-50-1.8e+27	0.57
**ThPD1**	0.27	0.011-6.5	0.42	0.1	0.00055-18	0.39
**Treg**	2.10E-15	1.1e-30-3.9	0.06	4.30E-18	2.7e-41-680000	0.14
**TregPD1**	3.50E-06	3.5e-15-3600	0.24	5.90E-15	2.7e-33-13000	0.13

Single-Marker

% of Total	DFS			OS		
**Stroma**	**HR**	**95% CI for HR**	**p.value**	**HR**	**95% CI for HR**	**p.value**
**CD3**	0.87	0.71-1.1	0.16	0.94	0.75-1.2	0.56
**CD4**	0.87	0.74-1	0.1	0.86	0.69-1.1	0.21
**CD8**	0.76	0.57-1	0.07	0.83	0.58-1.2	0.3
**FOXP3**	0.78	0.56-1.1	0.14	0.67	0.39-1.2	0.15
**PD1**	0.5	0.2-1.2	0.12	0.83	0.33-2.1	0.7
**Epithelia-associated**						
**CD3**	0.64	0.29-1.4	0.27	0.75	0.28-2	0.56
**CD4**	0.52	0.082-3.3	0.49	0.027	0.00015-4.8	0.17
**CD8**	0.34	0.1-1.1	0.081	0.36	0.071-1.8	0.21
**FOXP3**	0.00018	2.4e-09-13	0.13	6.10E-10	3.7e-20-10	0.077
**PD1**	0.052	0.0023-1.2	0.064	0.13	0.0035-4.9	0.27

Supplementary Table 4: Hazard Ratios for single-marker and multi-marker T cell subsets from immune hot-spot

Multi-Marker

% of Total	DFS			OS		
**Stroma**	**HR**	**95% CI for HR**	**p.value**	**HR**	**95% CI for HR**	**p.value**
**Tc**	0.84	0.58-1.2	0.37	0.83	0.47-1.4	0.51
**TcPD1**	0.67	0.33-1.4	0.27	1	0.49-2.1	0.95
**Th**	0.59	0.17-2	0.4	0.31	0.026-3.6	0.35
**ThPD1**	0.79	0.46-1.4	0.39	0.8	0.4-1.6	0.54
**Treg**	0.52	0.27-0.98	0.042*	0.25	0.061-1	0.052
**TregPD1**	0.59	0.24-1.5	0.25	0.8	0.28-2.3	0.68
**Epithelia-associated**						
**Tc**	0.39	0.059-2.6	0.33	0.22	0.0099-4.9	0.34
**TcPD1**	0.32	0.063-1.6	0.16	0.33	0.035-3.1	0.33
**Th**	3300	5.2e-06-2.1e+12	0.43	0.052	4.2e-19-6.5e+15	0.88
**ThPD1**	0.9	0.27-3	0.86	0.5	0.05-4.9	0.55
**Treg**	6.90E-08	2e-17-240	0.14	1.70E-13	7.3e-32-410000	0.17
**TregPD1**	0.71	1.3e-05-40000	0.95	7.50E-11	2.5e-24-2300	0.14

Single-Marker

% of Total	DFS			OS		
**Stroma**	**HR**	**95% CI for HR**	**p.value**	**HR**	**95% CI for HR**	**p.value**
**CD3**	0.91	0.8-1	0.17	0.94	0.8-1.1	0.45
**CD4**	0.92	0.83-1	0.11	0.89	0.76-1	0.13
**CD8**	0.86	0.71-1	0.12	0.83	0.62-1.1	0.2
**FOXP3**	0.77	0.59-1	0.052	0.66	0.43-1	0.066
**PD1**	0.7	0.41-1.2	0.21	0.88	0.47-1.6	0.68
**Epithelia-associated**						
**CD3**	1	0.73-1.5	0.87	1.1	0.74-1.7	0.59
**CD4**	0.91	0.44-1.9	0.81	0.38	0.044-3.4	0.39
**CD8**	0.45	0.19-1.1	0.085	0.3	0.064-1.4	0.13
**FOXP3**	0.11	0.00019-61	0.49	7.90E-05	7.6e-11-81	0.18
**PD1**	0.2	0.029-1.3	0.097	0.23	0.018-2.8	0.25

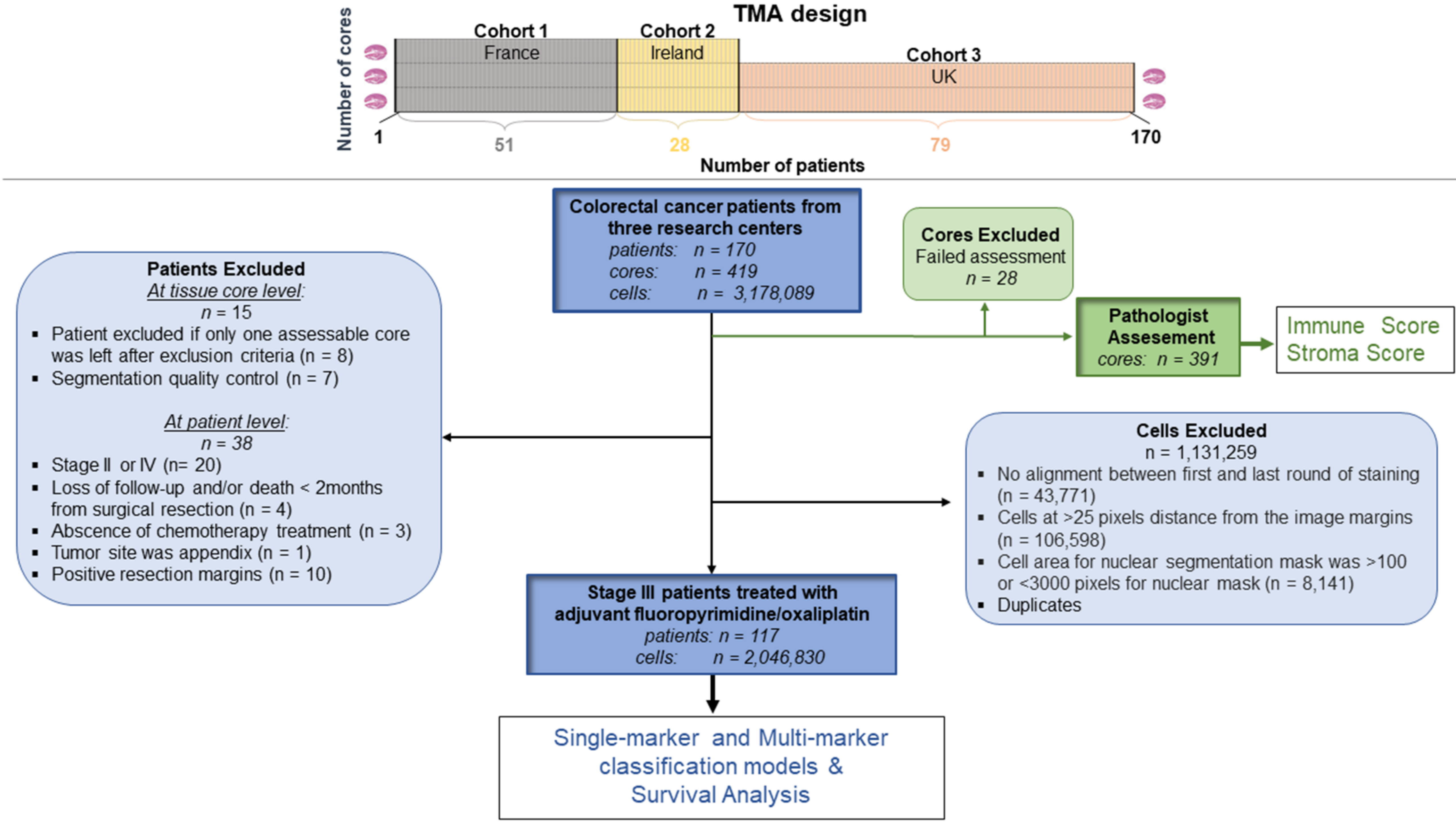

Supplementary Figure 1. CONSORT Flowchart: green boxes: participants for comparison of Pathologist's scores with Cell DIVE scores, blue boxes: analysis for association of single- and multi-marker classification with survival.

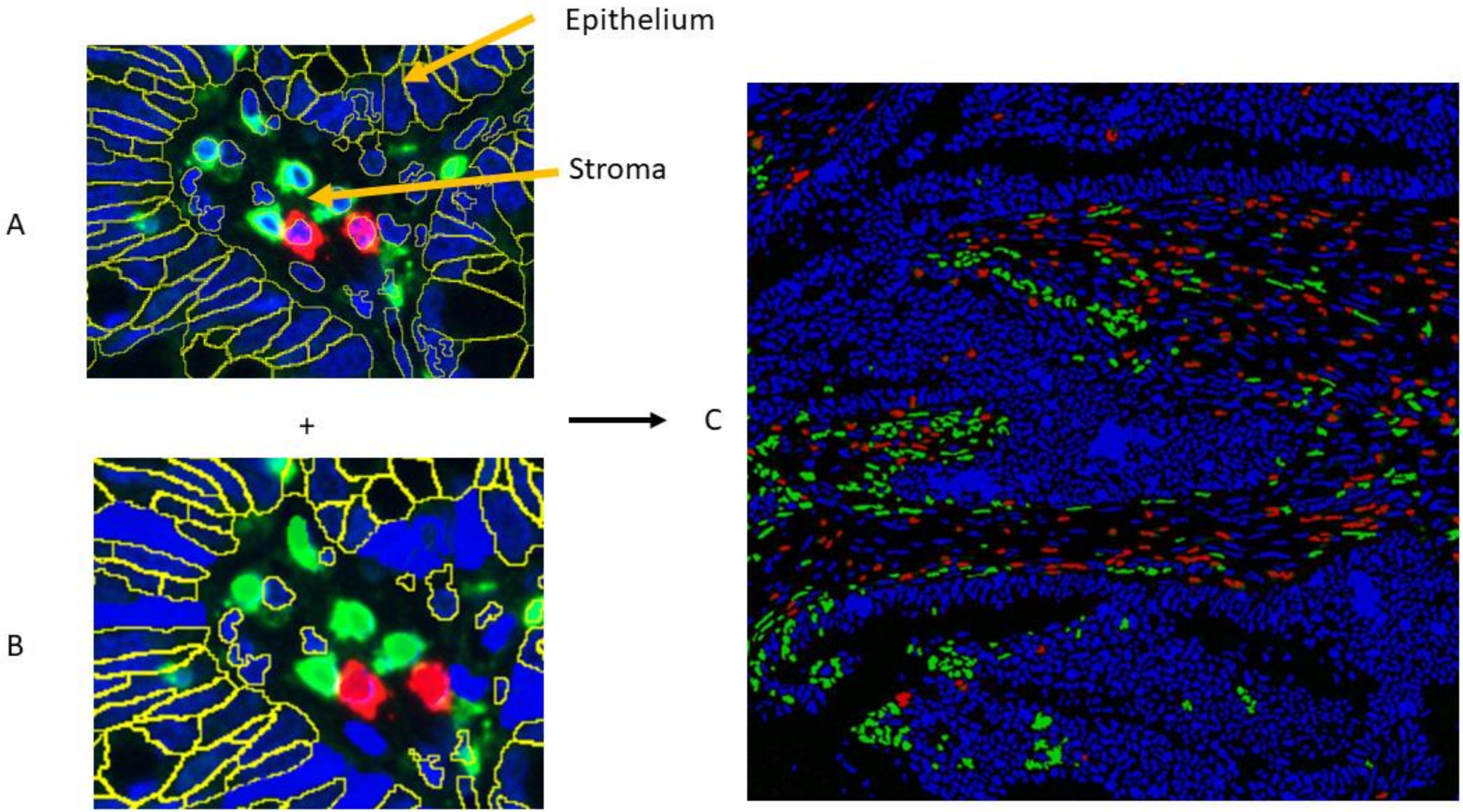

Supplementary Figure 2. Example images from the classification workflow. **(A)** Image showing generated epithelial and stromal nuclei segmentation masks overlaid on DAPI (blue), CD3 (green) and CD20 (red) images. **(B)** Annotations for a model classifying CD3 (green), CD20 (red), and negative cells (blue). **(C)** Predicted cell classes for a representative sample using the same color scheme as annotations. For multi-marker classification, the same cell is annotated based on multiple marker expression. This process is further described in Santamaria-Pang et al 2017[51].

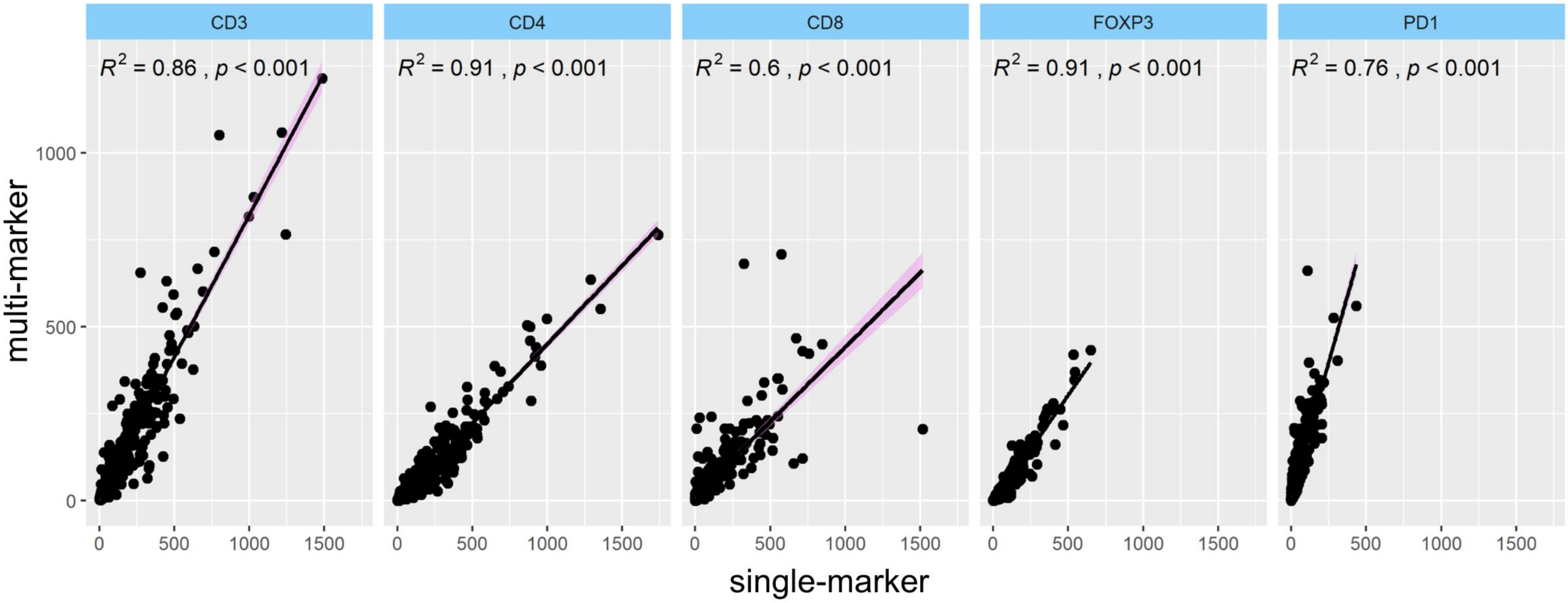

Supplementary Figure 3. Pearson Correlations between cell counts for single-marker model (x-axis) and multi-marker (y-axis) classification. **CD3**: CD3 vs Tc+/TcPD1+/Th+/ThPD1+/Treg+/TregPD1, **CD4**: CD4 vs Th+/ThPD1+/Treg+/TregPD1, **CD8**: CD8 vs Tc+/TcPD1, **FOXP3**: FOXP3 vs Treg+/TregPD1, **PD1**: PD1 vs TcPD1+/ThPD1+/TregPD1. The average counts of % of Total per patient were used for the analysis. R^2 for CD3, CD4, CD8, FOXP3 and PD1 were 0.86, 0.91, 0.6, 1.91 and 0.76 respectively. P was <0.001 for all combinations.

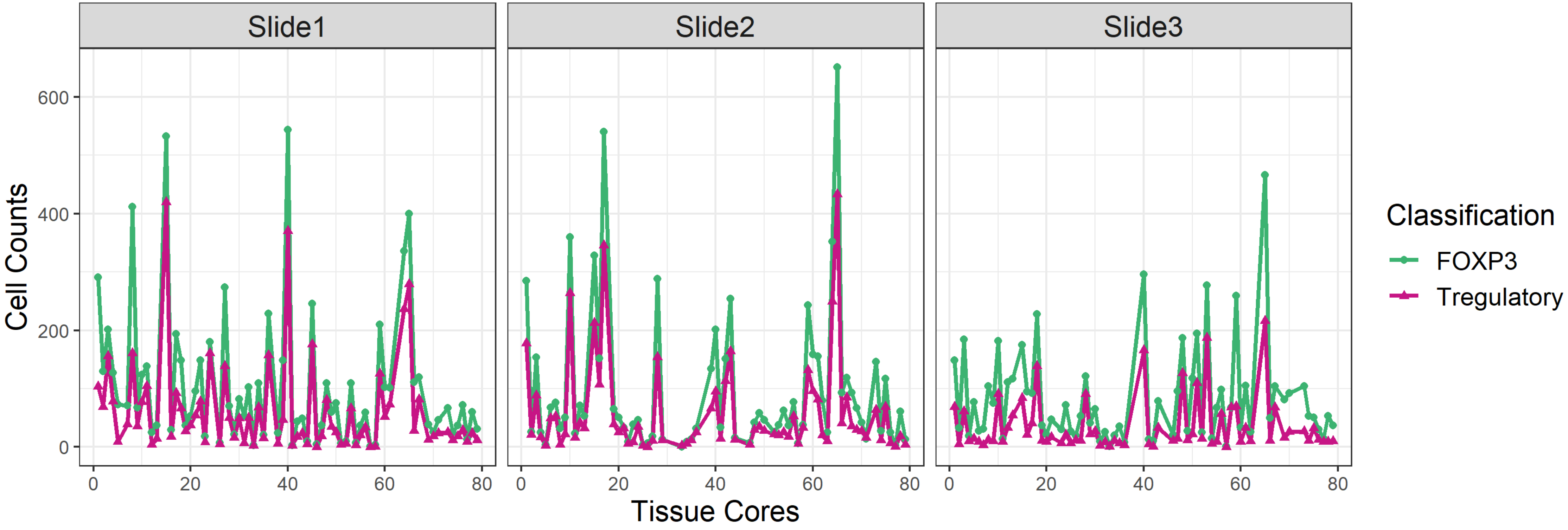

Supplementary Figure 4. Representative example of counts for the 79 patients with 3 cores (Slides 1-3) and comparison of single marker FOXP3 (green) vs multi-marker Treg (TregPD1- and TregPD1+) (purple) counts, per core. As FOXP3 is a marker for Treg cells, single marker FOXP3 counts are similar to the multi-marker Treg CD3+/CD4+/FOXP3+/(PD1+/-) counts.

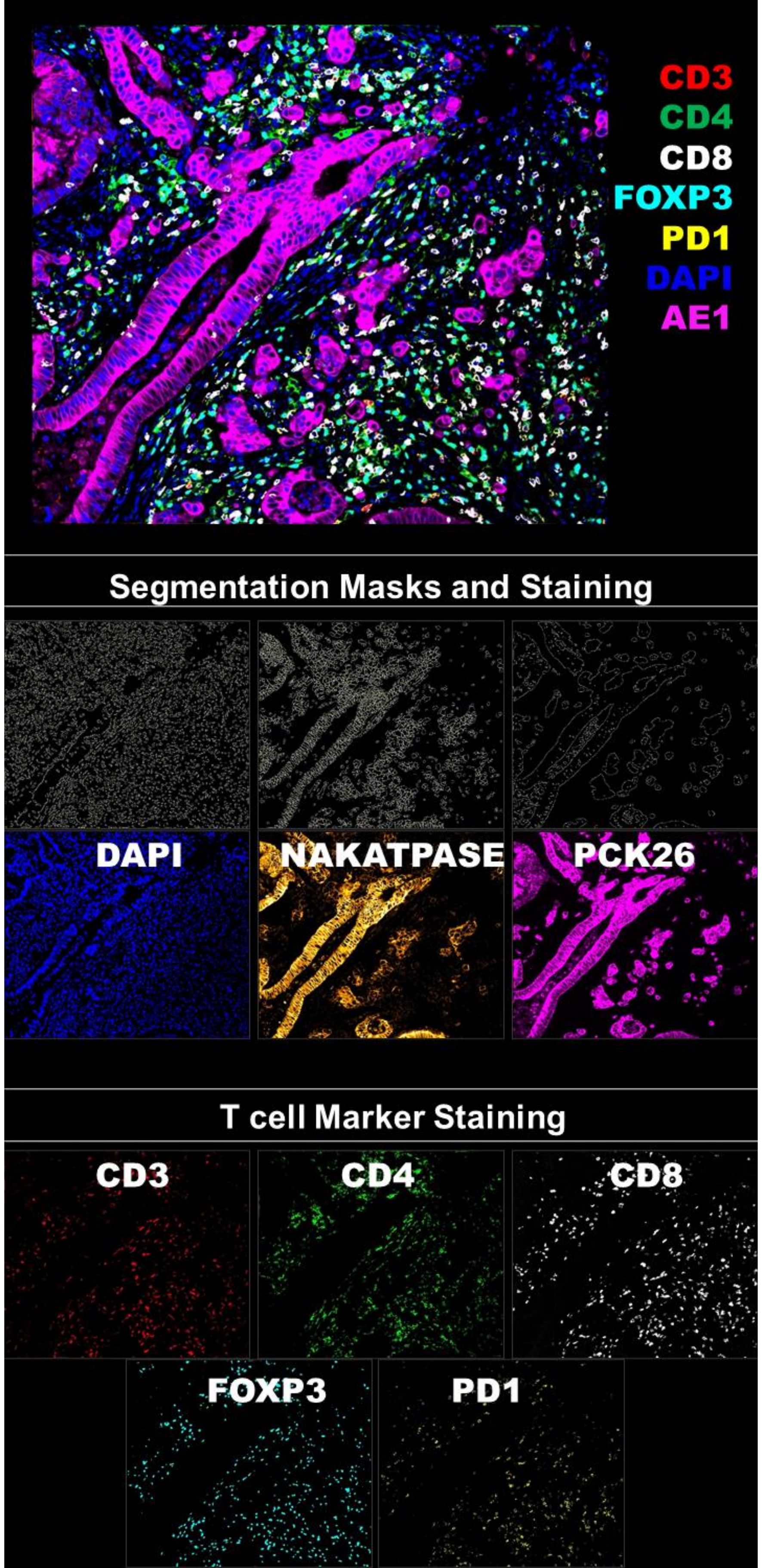

Supplementary Figure 5. Panel of representative multiplexed image with the corresponding segmentation masks and individual staining for each antibody used for T cell classification and segmentation in this study. For segmentation, DAPI, *Na K ATPase and PCD26 were used for the nuclear, membrane and epithelial mask, respectively. For the classification workflows, CD3, CD4, CD8, FOXP3 and PD1 markers were used.*

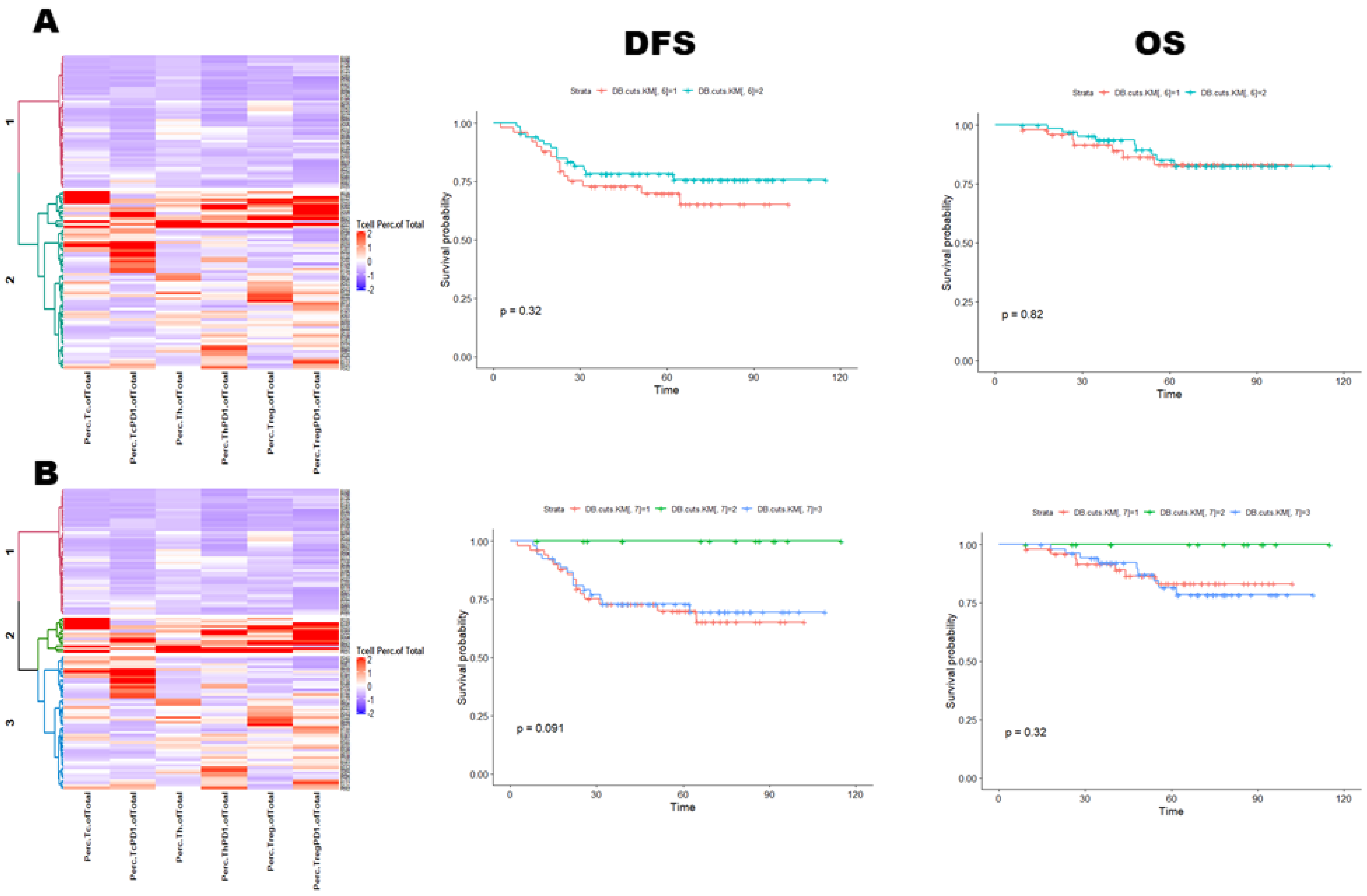

Supplementary Figure 6. Heatmaps showing separation and clustering of patients based on % T cells of Total T cell subtypes in tumor cores. Clusters based on the Ward.D agglomerative clustering method with Euclidean correlation distance measure. Kaplan-Meier curves color-coded for the corresponding 2 **(A)** and 3 **(B)** clusters of patients, demonstrating univariate survival analysis for DFS and OS. Differences in Kaplan–Meier survival curves are presented as log-rank p value.

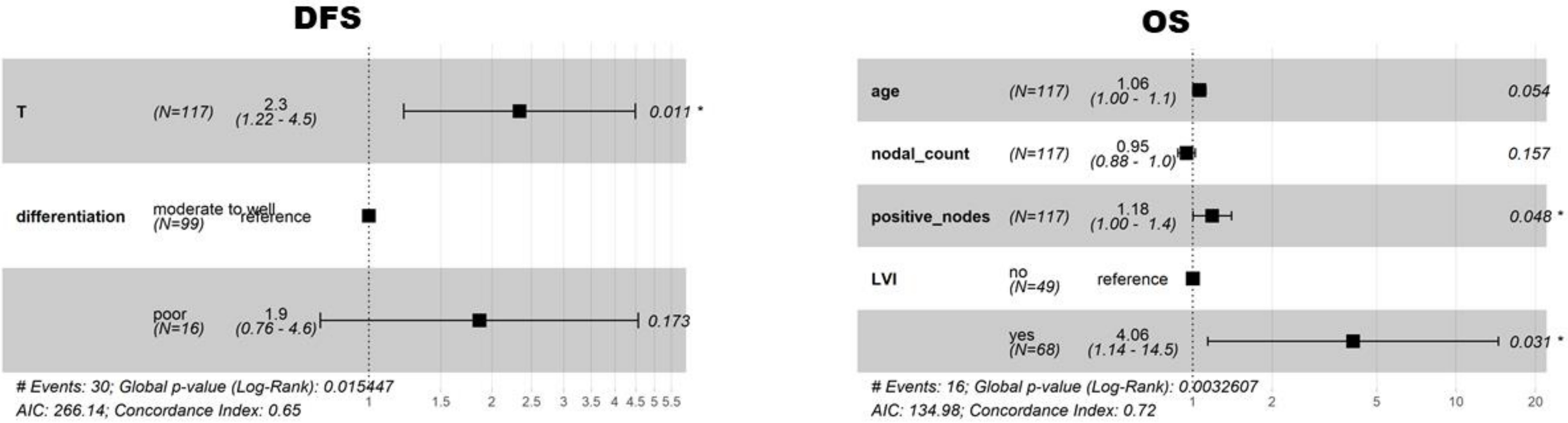

Supplementary Figure 7. Forest plots of multivariate Cox proportional hazards models for clinical variables after backward elimination. HRs, 95% CIs, and p values from likelihood ratio tests from univariate Cox proportional hazards models were calculated to explore the associations between the percent of total classified cells with the risks of recurrence (DFS) and death (OS). The clinical variables included in the model were: T, N, age, sex, nodal count, positive nodes, differentiation, lymphovascular invasion (LVI).

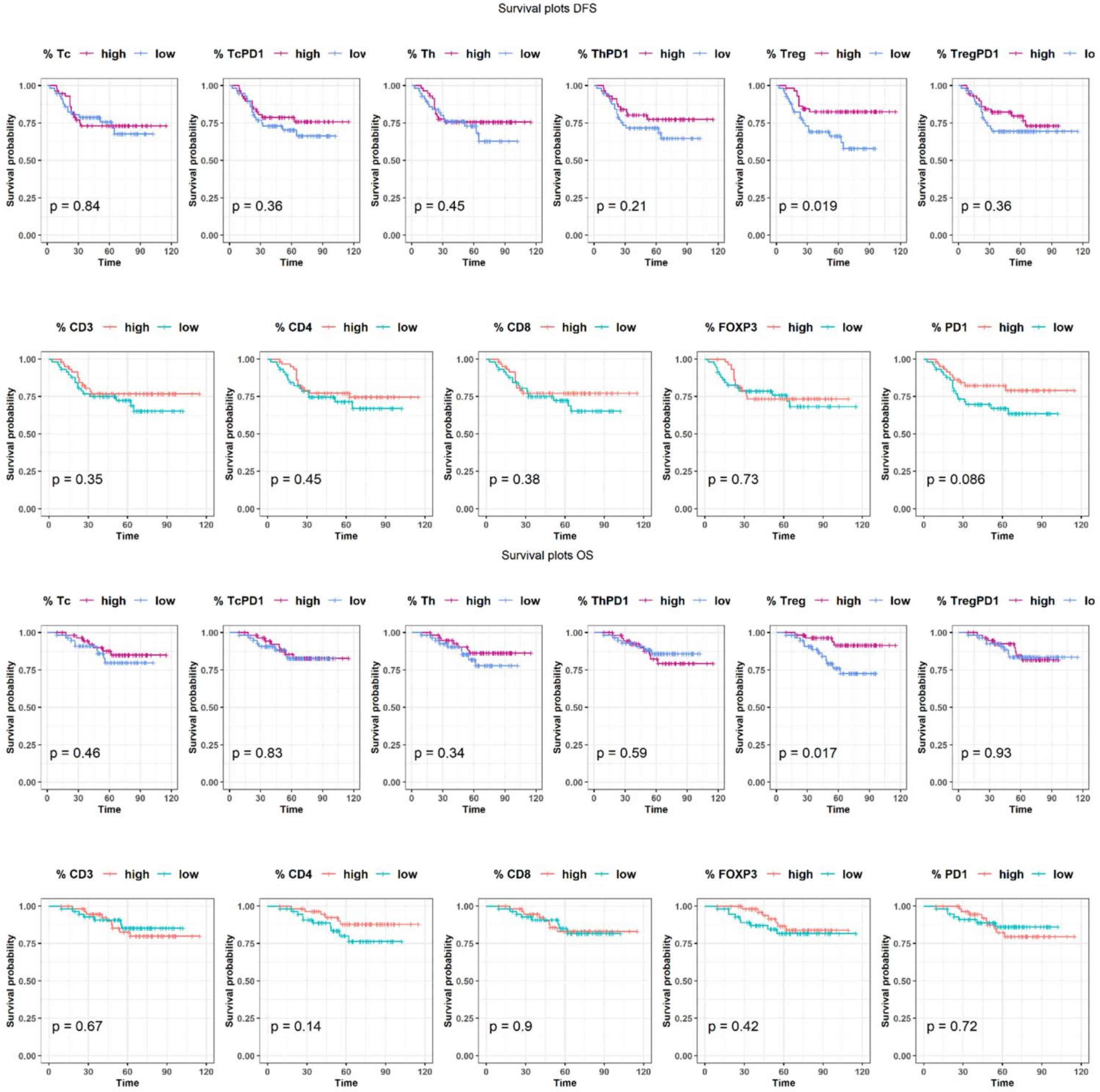

Supplementary Figure 8. Univariate survival analysis using Kaplan-Meier curves for single-marker and multi-marker T cell subtypes using the average percentage of total T cells for each patient (average of each patient's cores). The patients were dichotomized using the median as cut-off for DFS and OS. For multi-marker subtypes the groups were color-coded: high (red) – low (blue) and for the single-marker subtypes: high (orange) – low (green). The top two panels represent DFS and the lower two panels OS. Differences in Kaplan–Meier survival curves are presented as log- rank P value.

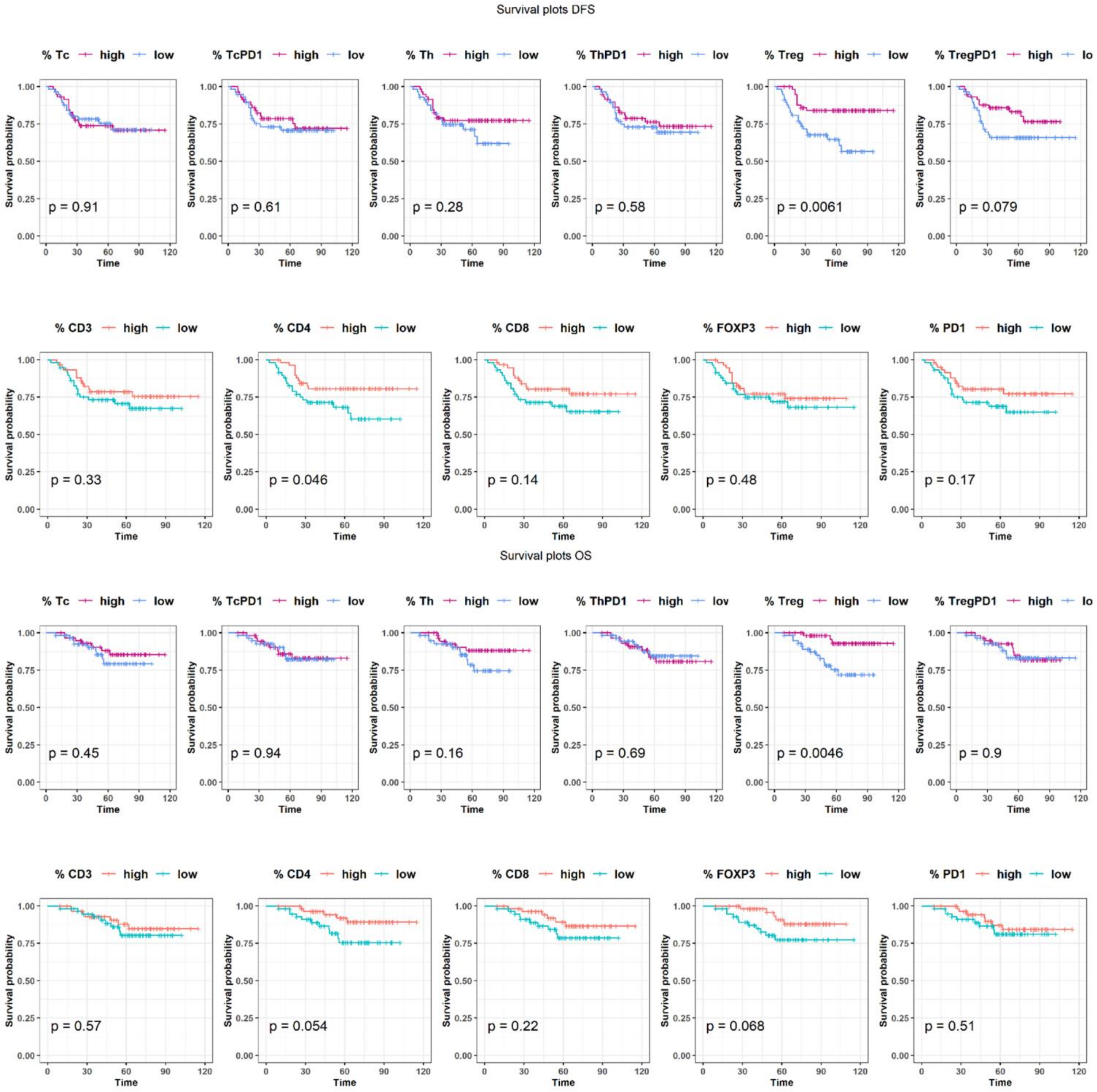

Supplementary Figure 9. Univariate survival analysis using Kaplan-Meier curves for single-marker and multi-marker T cell subtypes as percentage of total for immune hot-spot cores. The patients were dichotomized using the median as cut-off for DFS and OS. For multi-marker subtypes the groups were color-coded: high (red) – low (blue) and for the single-marker subtypes: high (orange) – low (green). The top two panels represent DFS and the lower two panels OS. Differences in Kaplan–Meier survival curves are presented as log- rank P value.
